# Secondary metabolites in grapevine: crosstalk of transcriptional, metabolic and hormonal signals controlling stress defence responses in berries and vegetative organs

**DOI:** 10.3389/fpls.2023.1124298

**Published:** 2023-06-19

**Authors:** Alessandra Ferrandino, Chiara Pagliarani, Eva Pilar Pérez-Álvarez

**Affiliations:** ^1^ Department of Agricultural, Forest and Food Sciences (DISAFA), University of Torino, Grugliasco, Italy; ^2^ National Research Council, Institute for Sustainable Plant Protection (CNR-IPSP), Torino, Italy; ^3^ Grupo VIENAP. Finca La Grajera, Instituto de Ciencias de la Vid y del Vino (ICVV), Logroño, La Rioja, Spain

**Keywords:** secondary metabolism, *Vitis vinifera* L., interaction with pathogens and environment, transcriptional and post-transcriptional regulation, epigenetic

## Abstract

Abiotic stresses, such as temperature, heat waves, water limitation, solar radiation and the increase in atmospheric CO_2_ concentration, significantly influence the accumulation of secondary metabolites in grapevine berries at different developmental stages, and in vegetative organs. Transcriptional reprogramming, miRNAs, epigenetic marks and hormonal crosstalk regulate the secondary metabolism of berries, mainly the accumulation of phenylpropanoids and of volatile organic compounds (VOCs). Currently, the biological mechanisms that control the plastic response of grapevine cultivars to environmental stress or that occur during berry ripening have been extensively studied in many worlds viticultural areas, in different cultivars and in vines grown under various agronomic managements. A novel frontier in the study of these mechanisms is the involvement of miRNAs whose target transcripts encode enzymes of the flavonoid biosynthetic pathway. Some miRNA-mediated regulatory cascades, post-transcriptionally control key MYB transcription factors, showing, for example, a role in influencing the anthocyanin accumulation in response to UV-B light during berry ripening. DNA methylation profiles partially affect the berry transcriptome plasticity of different grapevine cultivars, contributing to the modulation of berry qualitative traits. Numerous hormones (such as abscisic and jasmomic acids, strigolactones, gibberellins, auxins, cytokynins and ethylene) are involved in triggering the vine response to abiotic and biotic stress factors. Through specific signaling cascades, hormones mediate the accumulation of antioxidants that contribute to the quality of the berry and that intervene in the grapevine defense processes, highlighting that the grapevine response to stressors can be similar in different grapevine organs. The expression of genes responsible for hormone biosynthesis is largely modulated by stress conditions, thus resulting in the numeourous interactions between grapevine and the surrounding environment.

## 1 Introduction

Climate change-associated stresses, such as frequent drought events and heat waves during the growing season, have been seriously impacting the viticultural sector worldwide (Horton et al., 2016; Wolkovich et al., 2018). Unexpected long, hot, and dry periods during summer cause intensification of some pest and pathogen spread (Corredor-Moreno and Saunders, 2020), with dramatic effect on potential vine yield and quality. In the last few years, incidence of some fungal/oomycete-associated diseases significantly increased due to climate alterations (Gullino et al., 2018). The analysis of the impact of these phenomena led some climate-based predictive models to project a decline in the suitability of traditional wine growing regions (Hannah et al., 2013). Such predictions, however, do not consider potential buffering effects due to the high intraspecific genetic variability in grapevine and to grapevine phenotypic plasticity. The ability of grapevine varieties and clones to respond to changing conditions by shaping their phenotype (Sultan, 2010; Dal Santo et al., 2018) could indeed limit the forecasted decline of viticultural areas (Wolkovich et al., 2018; Morales-Castilla et al., 2020). Additionally, mitigating the effects of climate change also relies on the adoption of specific agricultural practices like optimizing rootstock choice. Through such practices, the grapevine’s capability to recover from water stress can be exploited with retention if not improvement of the berries’ qualitative traits (Patono et al., 2022). Global warming negative effects on grapevine phenology could be limited by exploiting the great intraspecific variability of wine grapes that exists at the cultivar/clone level and that deeply influences the genotype physiological responses to environmental cues like drought (Bota et al., 2016; van Leeuwen et al., 2019). Moreover, the use of late-ripening and drought-resistant cultivars and/or grafting onto drought-tolerant rootstocks represent cost-effective and environmentally friendly strategies for improving vine resilience to water deficit and high temperatures, thereby ensuring the production of high-quality wines. The development of cutting-edge agronomic practices for enhancing grapevine tolerance to stress is therefore a strict requirement that inevitably calls for an in-depth knowledge of the biological mechanisms controlling the vine metabolic responses to climate change effects (e.g. accumulation of defence- and quality-associated secondary metabolites). A comprehensive understanding of the genetic regulation of grape berry secondary metabolism in response to altered climatic conditions is indeed crucial to identifying the drivers of fruit quality. The challenge to filling the knowledge gaps on such themes is deeply felt among both the scientific community and viticulturists, as demonstrated by the increasing number of studies addressing the analysis of berry secondary metabolic composition in different grapevine genotypes exposed to abiotic stresses. The main achievements in this research field have been outlined in recent reviews. Gouot et al. (2019) provide a closer look into the impact of increasing temperatures on grape flavonoid metabolism, and Rienth et al. (2021a) discuss the influence of the main abiotic stresses (high temperature, drought, excessive UV-B radiation, high CO_2_ concentrations) on shifts in the amounts and partitioning of berry secondary metabolites.

Besides their well-known contribution to berry ripening, secondary metabolites are important players in plant defence. Nevertheless, their role in biotic stress response has been studied only recently in *Vitis* spp. The analysis of grapevine responses to pathogen attacks in relation to the modulation of secondary metabolic pathways involves both berries and vegetative tissues. In fact, leaves, stems, wood, and roots are the organs where many diseases spread, such as powdery and downy mildews, wood diseases, viruses, and bacteria. Based on this context, the present review will discuss the effect of the main abiotic and biotic stresses on secondary metabolic processes occurring in berries and vegetative organs by detailing how the accumulation of individual classes of compounds is mediated by molecular signalling cascades such as those triggered by short RNAs (sRNAs), epigenetic modifications and hormonal crosstalk. The impact that the genotype × environment interaction exerts in orchestrating plant defence-signalling cascades will also be considered. Finally, attention will be given to highlight those secondary metabolic changes that underlie tolerance/resistance mechanisms to environmental cues, particularly to some grapevine pathogens (i.e. *Botrytis cinerea*, Grapevine Trunk Diseases and phytoplasmas). From the short to medium-term perspective, the identification of the biological processes at the basis of grapevine resilience to stress should indeed provide precious information for optimising sustainable disease protection practices in viticulture.

## 2 Molecular and hormonal crosstalk modulating the grape berry secondary metabolism in response to abiotic stress

To disclose the complexity of stress-dependent molecular players either driving or inhibiting the accumulation of secondary metabolites, it is pivotal to understand how these metabolic pathways are under genetic control. Notably, in the last years, research in this field has been boosted owing to the great advances made in high-throughput sequencing techniques, coupled with the availability of the reference grapevine genome (Jaillon et al., 2007) and the release of the genome sequences of several *Vitis vinifera* cultivars and rootstocks (http://www.grapegenomics.com/; Minio and Cantu, 2022). Recently, Rienth et al. (2021a) provided an in-depth overview on the changes in the expression of key genes regulating the biosynthesis and mobilisation of grapevine secondary metabolites in response to different abiotic stresses. To avoid overlapping with this recent review, in this section we focus on the molecular and hormonal crosstalk beneath secondary metabolism in grapevine berries. We detail: i) post-transcriptional and epigenetic regulation; ii) hormonal cross talk regulation; and iii) genotype × environment interaction effects on berry secondary metabolite composition.

### 2.1 Stress-induced post-transcriptional and epigenetic changes regulating berry secondary metabolism

In the last decade, several studies attempted to dissect the whole transcriptional reprogramming associated with modification of secondary metabolism in response to stress. Unlike other crop species (Jogawat et al., 2021), post-transcriptional regulatory mechanisms linked to those responses, such as those mediated by small RNAs (sRNAs), have been rarely considered in grapevine.

sRNAs represent tiny signalling molecules that modulate a *plethora* of plant growth and developmental processes by transcriptionally and post-transcriptionally regulating their target genes. Emerging experimental evidence has proven that the synergic interplay among sRNAs and hormone signalling pathways is crucial for driving plant responses to multiple environmental changes (Li et al., 2020). Of note, sRNAs, and mainly microRNAs (miRNAs), respond to, or exhibit overlapping regulatory activities with those of phytohormones (Curaba et al., 2014). As a result, miRNAs are often referred to as RNA hormones. Intriguingly, hormones and sRNAs can move cell-to-cell or systemically within the plant vascular tissues, thereby controlling their targets over long distance (Pagliarani and Gambino, 2019). Pinto et al. (2016) analysed the distribution of small RNAs in the berries of Cabernet Sauvignon and Sangiovese, in order to compare the changes occurring in response to three different environments. The study disclosed key relationships between the differential expression of specific miRNAs and some genes involved in secondary metabolism, leading to the identification of two novel miRNAs whose target transcripts encoded enzymes of the flavonoid biosynthetic branch. It also emerged that environmental and vineyard-management conditions did affect the transcription of miRNA-producing gene *loci*, although, patterns of miRNA accumulation were predominantly influenced by the cultivar genotype and by the specific berry developmental stage. A close overview on the effect of abiotic stress factors on the modulation of small RNAs regulating secondary metabolism in grape berries has recently been provided by Sunitha et al. (2019). In a study of Cabernet Sauvignon vines grown both under greenhouse and field conditions, the researchers analysed the impact of UV-B radiation on the abundance of miRNAs and of phased small-interfering-RNA (phasi-RNAs)-producing *loci* during the fruit development and berry ripening periods. They identified novel and conserved miRNA-mediated regulatory cascades (including the autoregulatory loop involving miR828-TAS4) that by post-transcriptionally controlling key MYB transcription factors, shape the accumulation of anthocyanins in response to UV-B light during berry ripening (Sunitha et al., 2019).

Another intriguing and still little investigated research matter is represented by the analysis of epigenetic marks underpinning the modulation of berry secondary metabolic pathways in response to environmental stressors. Although important breakthroughs in the understanding of epigenetic phenomena controlling the plant adaptation to drought and temperature stress have been made in many economically important crops (Varotto et al., 2020), very little information is available in grapevine (Fortes and Gallusci, 2017). A mechanistic link between epigenetic modifications and accumulation of specific berry secondary metabolites has not been provided so far. Nevertheless, it was shown that changes in DNA methylation profiles could partially affect the berry transcriptome plasticity of different grapevine cultivars (Dal Santo et al., 2018), and in turn contribute to the modulation of fruit and wine quality traits related to terroir (Xi et al., 2017). Further investigation on the role of stress-responsive sRNAs and epigenetic modifications in the control of berry secondary metabolic pathways will also be valuable to gain more information on the activation of specific defence processes. For instance, a still to be deepened subject in grapevine is the study of the synergy between sRNA/epigenetic-based regulatory networks and other overlapping signalling routes, such as those dependent on hormones and those dependent on the accumulation of defence and quality-associated molecules.

### 2.2 Hormonal cross talk regulating the berry secondary metabolism upon stress

Transcriptional reprogramming events can thoroughly modify hormone metabolism and signalling. Moreover, molecular players and hormone crosstalk pathways synergically work to reach a tight control on secondary metabolism during berry development (Kuhn et al., 2014). Notably, hormone amounts do vary according to the vine stress response (Ferrandino and Lovisolo, 2014) and to the specific berry developmental stage (Kuhn et al., 2014). The progressive hormone accumulation in the berry serves as a trigger for the synthesis of secondary metabolites, such as the case of the ABA-mediated activation of the anthocyanin biosynthesis at véraison (Castellarin et al., 2016). Available literature also clearly attested the predominant role that diverse hormone signalling cascades exert on the regulation of berry secondary metabolism upon stress conditions (Fortes et al., 2015). Even though many papers described the effects exerted by temperature, heat flash, light intensity, and quality on grapevine secondary metabolism, much less is known about the hormonal crosstalk behind these events. A recent study by Min Liu and co-workers (2020) revealed that grapevine response to heat stress and heat acclimation treatments drives an intense transcriptional reprogramming involving the overexpression of 25 heat-shock proteins, 11 antioxidases and 31 transcription factors. Notably, those transcriptional modifications occur in parallel with changes in the sugar-ABA signalling cross talk. Particularly, it was observed that following acclimation to heat stress, grapevine genes encoding sucrose phosphate synthases, sucrose synthases and invertases were transcriptionally inhibited, thereby leading to an overall decrease in the cell sucrose amounts. Such condition represented the trigger for the activation of the ABA signaling pathway and the downstream induction of stress-responsive genes (Min Liu et al., 2020). This picture of molecular and biochemical interactive networks suggests a shift in the growth-defence trade off balance, which may divert the vine energy metabolism to establish stress defence reactions associated with thermotolerance. A stress-induced shift from primary to secondary metabolic reactions is also a hallmark of grapevine response to high light exposure. Light quality and intensity (high light in particular) conditions were indeed shown to favour the accumulation of antioxidant molecules and secondary metabolites in grape berries at the expense of carbohydrate metabolism. Such events were found to occur through extensive modifications of grapevine transcriptome, which mainly rely on the upregulation of defence related genes, including those involved in the production of anthocyanins, flavonols and carotenoids (du Plessis et al., 2017). Additionally, the overexpression of the 9-cis-epoxycarotenoid dioxygenase encoding gene (*VvNCED*) directly linked to ABA biosynthesis suggested an increase in ABA concentration and a consequent activation of the ABA signalling pathways that may, in turn, promote grapevine adaptation to high light intensity. It is thus clear that, among all phytohormones, ABA plays the major role in the control of berry phenology and stress perception (Kuhn et al., 2014; Pilati et al., 2017). Moreover, it has long been demonstrated in different cultivars that exogenous ABA application can mediate the accumulation of phenolic components in the berries (Quiroga et al., 2012; Xi et al., 2013; Luan et al., 2014) by activating transcription of key genes involved in the flavonoid pathway, such as phenylalanine ammonia lyase, chalcone synthase, glutathione S-transferase and MyB-related transcription factors (Koyama et al., 2010). Additionally, exogenous ABA treatment of berries is an effective strategy for managing the uncoupling of technological and phenolic fruit ripening in wine grape varieties due to the imbalance between sugars and anthocyanin concentrations upon challenging environmental conditions (Wang et al., 2021). Application of exogenous ABA in the presence of abiotic factors such as UV-B light and water deficit was shown not only to promote the biosynthesis and accumulation of anthocyanins, but also to increase the amounts of piceid and *trans*-resveratrol in the berry skin of cv Malbec. The latter result was mainly observed when ABA application was performed in concomitance with elevated UV-B radiation. Unlike phenylpropanoids, volatile aromatic components were poorly affected by the imposed ABA treatments (Alonso et al., 2021).

Further research efforts have been made to unlock the complexity of interconnected networks of hormones that shape the grapevine metabolism in response to the environment. For instance, it was proposed that other carotenoid-derived hormones, like strigolactones (SLs), could interact with ABA regulatory pathways, particularly with the ABA-mediated anthocyanin accumulation in the berries at véraison (Ferrero et al., 2018). Grape berries co-treated with ABA and the synthetic strigolactone analogue GR24 experienced a delay in anthocyanin accumulation at véraison, which is supported by the down-regulation of the anthocyanin-related genes *VvMybA1* and *VvUFGT*. The ABA-GR24 co-treatment also negatively affected ABA concentration in the berry. Interestingly, this condition was not achieved by inhibiting the transcription of ABA biosynthetic genes (i.e. *VvNCED*), but rather it depended on the activation of the hormone catabolic pathway (e.g. ABA-8’-hydroxylases) and on perturbation of the ABA cellular/apoplastic delivery (Ferrero et al., 2018). These findings may thus call for a role of SLs within the interactive network of molecular players that regulate ABA accumulation and signalling.

Moreover, increasing evidence shows that another crucial crosstalk in the regulation of the plant’s response to abiotic stress is established between ABA and jasmonate (JA) signalling pathways (Yang et al., 2019). Like ABA, the plant’s response to JA elicits different defence processes, including detoxyfication of reactive oxygen species and increase of osmoprotectant concentrations at the cellular level. In different crop species it was demonstrated that the two hormones operate in a synergistic way by reciprocally activating the molecular effectors involved in their biosynthetic and signalling routes (Kim et al., 2021). For instance, the first step of the ABA-JA crosstalk involves the dynamic interaction between the ABA-induced PYRABACTIN RESISTANCE1-Like protein (PYL) and the JA-based JAZ–MYC2 module, responsible for the signalling cascade that coordinates the balance between the activation of either plant growth or defence mechanisms (Yang et al., 2019). Consistently, experiments conducted on *V. vinifera* cv Summer Black vines attested that endogenous levels of both ABA and JA strongly increased in stressed leaves. Such metabolic changes occurred concomitantly with a steep upregulation of many transcripts encoding the defence-associated proteins that typically participate in plant immunity, such as heat shock proteins, MAP kinases, and WRKY transcription factors. In parallel, molecular effectors of the ABA and JA signalling pathways, like phosphatase 2C, calmodulin (CAL), calcineurin B like proteins (CBL), and jasmonate ZIM-domain proteins (JAZ) were also overexpressed (Haider et al., 2017). It must be noted that some of the observed transcriptional changes, such as the increase in CAL and CBL mRNAs, also indicated an overlapping with other stress-responsive signals, particularly the Ca^2+-^based ones, thus adding a further level of complexity into the molecular regulatory networks that control the plant’s response to abiotic stress.

Auxin-mediated molecular signals, on the other hand, are antagonistic to those induced by ABA. The analysis of the crosstalk between auxin and ABA highlighted a tight coordination occurring among the two hormonal pathways and sugars (sucrose) that is crucial for priming berry development. For instance, while ABA and sucrose treatments on the cultivar Fujiminori berries triggered fruit ripening processes, the application of IAA (indolacetic acid) delayed them, negatively affecting sugar and anthocyanin accumulation, fruit firmness, cell wall metabolism, and aroma spread. Such differences among the imposed treatments were underpinned at the transcriptome level by a strong transcriptional reprogramming of the main genes associated with berry ripening and flavonoid biosynthesis, like pectin esterases, polygalacturonases, pectate lyases, flavanone 3’-hydroxylases (*F3’H*), *CHS*, and *UFGT* (Jia et al., 2017). Analyses performed in Cabernet Sauvignon grapes following treatments with ABA and synthetic auxin further confirmed the effect of the ABA-auxin interaction on the regulation of anthocyanin and norisoprenoid biosynthesis, at both the metabolite and transcriptional levels (He et al., 2020; He et al., 2021). A tight interaction among hormone regulatory routes is also at the basis of the regulation of mono- and sesquiterpene biosynthesis in grapevine. Despite the accumulation of these molecules during the berry ripening and in response to stress cues was established to primarily depend on jasmonates (jasmonic acid, JA and methyl-jasmonate, MeJA) (Yang et al., 2019), a crosstalk between these and other ripening and stress-associated hormones, such as ABA, ethylene, and auxin, does also occur (Wang et al., 2022). Finally, treatments with exogenous ABA and gibberellins (GA3) were shown to differently affect the production and partitioning of antioxidants (proline and anthocyanins) and defence compounds (terpenes) in leaves, berries, and roots of cv Malbec plants (Murcia et al., 2017). These findings suggested that the interaction between ABA and gibberellin-dependent routes could promote the establishment of priming phenomena in the treated vines, prompting them to better cope with environmental stresses.

### 2.3 Grapevine adaptability to environmental stress: phenotypic plasticity and its effect on berry secondary metabolism

*Vitis* cultivars are adapted to grow in different mesoclimates (Morales-Castilla et al., 2020), making grapevine an interesting model species to study the genetic and molecular bases that underlie phenotypic plasticity (Dal Santo et al., 2013; Dal Santo et al., 2016; Dal Santo et al., 2018) in response to multiple environmental cues. The analysis of genotype-based transcriptional modifications influenced by the G × E interplay is crucial to understand the different regulation of metabolic pathways during berry ripening (Dal Santo et al., 2013; Massonnet et al., 2017). Existing literature takes into account the responses i) of the same cultivar to different growing areas (Cramer et al., 2014; Anesi et al., 2015; Dal Santo et al., 2016), ii) of several cultivars within the same growing area (Degu et al., 2014; Massonnet et al., 2017); and iii) of different genotypes cultivated across different viticultural sites and climate conditions (Ghan et al., 2015; Dal Santo et al., 2018). Anesi et al. (2015) analysed the whole transcriptome and metabolome of berries from a single Corvina clone grown in seven different vineyards during a 3-year trial period. The authors attested that some berry transcriptional and metabolic changes, like those resulting in the accumulation of flavonoids, anthocyanins, stilbenes and sesquiterpenes were significantly shaped by the *terroir* and this effect was maintained over several vintages. Conversely, the biosynthesis of other metabolic components, such as hydroxycinnamic acid derivatives and flavan-3-ols/procyanidins showed the least level of plastic response to environment (Anesi et al., 2015). Among the studies addressing the response of different cultivars to the same growing area, the one by Degu and coworkers (2014) was the first to combine data from targeted metabolomics and transcriptome sequencing with the goal to dissect changes occurring during ripening in the berry skin of Shiraz and Cabernet Sauvignon grown in the same semiarid environment. Notably, the outcomes of the study were maximised by the fact that two red-grape varieties used displayed opposite physiological behaviours to water deficit. The authors reported that along with ripening and water deficit progression, the regulation of berry development in Shiraz was based on a better coordination between primary and secondary metabolic signals with respect to Cabernet Sauvignon, and this resulted in a higher transcription of genes of the phenylpropanoid pathway. Such genotype-dependent features also led to increased production of stress-responsive secondary metabolites in Shiraz berries that, since véraison, showed higher amounts of piceid and of coumaroyl derivatives of anthocyanin than Cabernet Sauvignon. Those data, together with the exclusive upregulation of 19 hormone-related genes involved in abscisic acid (ABA) metabolism, suggested that physiological and metabolic responses to drought conditions were enhanced in Shiraz (Degu et al., 2014). Dal Santo and collaborators (2018) characterised plastic changes occurring across two vintages in the berry transcriptome and genome methylation landscapes of Cabernet Sauvignon and Sangiovese cultivated in three different geographical areas. From this comprehensive survey, it emerged that the G × E interaction exerts a key role in the modulation of genotype specific berry quality traits, such as those linked to the production of flavonoid and volatile organic compounds (VOCs). The importance of the genotype contribution in the regulation of berry secondary metabolism was further addressed by Gambino et al. (2021), who outlined the transcriptional changes occurring over ripening in berries of the red-grapes Nebbiolo and Barbera. Besides transcriptional modifications leading to distinct anthocyanin profiles typical of the considered varieties, the study highlighted, in Nebbiolo, a unique reprogramming of transcripts involved in the biosynthesis of defensive secondary metabolites (stilbene synthases), which, being activated regardless of pathogen or stress factor presence, suggested a more active basal defence metabolism in this cultivar. Studies of grapevine plastic metabolic responses to environmental conditions should also consider effects due to grapevine intra-varietal variability. Indeed, beside cultivar-based responses, clone-mediated signals can also affect the composition of berry secondary metabolites. The influence of clonal variability on secondary metabolism had been investigated at the analytical level, in different cultivars including Barbera, Cabernet Franc and Merlot (Ferrandino and Guidoni, 2010; van Leeuwen et al., 2013; Pantelić et al., 2016). On the contrary, clone × environment (C × E)-dependent changes underlying fruit quality were poorly studied at the molecular level. Research efforts in this direction were made by analysing in two vintages berry transcriptomic and metabolic data collected during ripening from three Nebbiolo clones grown in different vineyards (Pagliarani et al., 2019). Transcripts associated with sugar and hormone signalling cascades, that control anthocyanin and flavonoid biosynthesis downstream, were affected by the berry developmental stage. Conversely, genes linked to anthocyanin transport were expressed depending on the C × E interaction and according to changes in the berries’ anthocyanin partitioning. The study also indicated a strong effect of the vineyard sanitary status on the regulation of secondary metabolic pathways, leading to the production of antimicrobial compounds, such as phytoalexins (Pagliarani et al., 2019).

In terms of practical impact, overall information collected from G × E studies offers a valuable theoretical basis to orient the adoption of suited agronomic practices considering growing site and specific cultivar or clone. In parallel, it also provides scientific support for developing *ad hoc* breeding programs for selecting genotypes with improved tolerance to environmental threats.

## 3 Transcriptional reprogramming, hormonal crosstalk and specific molecule accumulation during pathogen pressure

Knowledge of the effects of the main biotic stresses on berry secondary metabolism has recently been revised in relation to fungal and viral pathogens (Rienth et al., 2021b). In the present review, we considered the effects of *Botrytis cinerea*, GTDs, and phytoplasma on grapevine secondary metabolism, focusing on the hormonal crosstalk involved in these three grapevine defence response patho-systems.

### 3.1 Botrytis cinerea (Bc)

**Figure 1:**
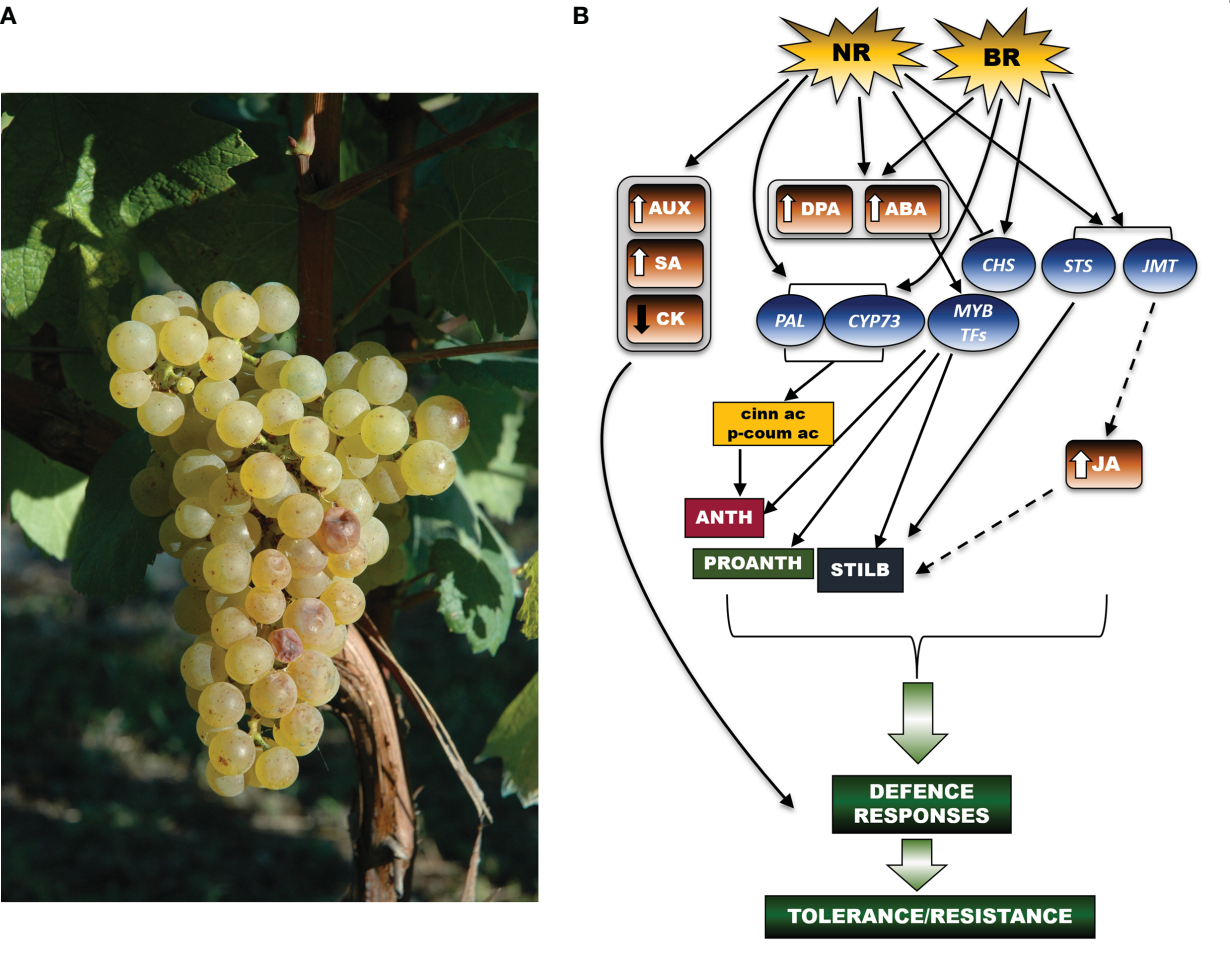
A schematic representation of the networks of hormonal and molecular signals regulating the secondary metabolism in the grapevine berry in response to *Botrytis cinerea*. **(A)** A grape bunch of *Vitis vinifera* cv Chardonnay showing very initial symptoms of *Botrytis cinerea* infection (credits: A. Ferrandino). **(B)** At the berry level, both NR (Noble Rot) and BR (Bunch Rot) infection induce an increase in DPA (dihydrophaseic acid) and ABA (abscisic acid) concentrations. ABA accumulation in turn activates the transcription of *MYB* genes regulating the biosynthesis of stilbenes, proanthocyanidins (PROANTH) and anthocyanins (ANTH). Response to NR also involves the upregulation of genes linked to auxin (AUX) and salicylic acid (SA) metabolism, and, in parallel the downregulation of the cytokinin (CK) metabolism. Following both NR and BR infection, the observed upregulation of jasmonate *O*-methyltransferase (*JMT)* transcripts suggests that jasmonate (JA)-mediated signals (stilbene = STILB biosynthesis) are also established. Furthermore, upon NR and BR development, genes of the phenylpropanoid pathway (*PAL*, phenylalanine ammonium lyase; *CYP73*, trans-cinnamate 4-monooxygenases) are turned on, downstream leading to the production of cinnamic acid (cinn ac) and p-coumaric acid (p-coum ac), which are intermediate bricks of the phenylpropanoid pathway. While *STS* isoforms are highly expressed in both NR and BR-infected berries, transcription of chalcone synthase (*CHS*) genes is differently regulated between NR (downregulation) and BR (upregulation). The set of displayed signals all converge to initiate defence mechanisms in turn leading to tolerance or resistance responses to the pathogen (as underlined by the curly green bracket). In the figure scheme, the arrows connecting the different signal effectors highlight activation of a specific molecular or metabolic pathway, whereas the blunt arrow points to inhibition of the downstream target(s). The dashed arrows display those signalling cascades that have been suggested but still need further experimental confirmation. The arrows beside the hormone acronyms refer to increase (↑) or decrease (↓) of accumulation. The thick green arrows point to the establishment of plant defence processes resulting from the activation of the upstream signalling pathway(s). Brown boxes refer to phytohormones, blue balloons indicate genes, while the rectangulars refer to metabolites.

Bc induces a substantial reprogramming of hormonal metabolism during grape berry ripening (**Figure 1**). A recent study reported a dramatic increase in ABA (up to 140-fold) and dihydrophaseic acid (DPA) in Noble Rot (NR)-infected berries (cv Furmint), but not in grey mold-infected Marselan berries (Pogány et al., 2022). ABA is a versatile player in the resistance against *Bc*. While on one hand this hormone contributes to *Botrytis* susceptibility in tomato leaves (Audenaert et al., 2002), on the other hand, and to a larger extent, it activates the transcription of MYB genes (AbuQamar et al., 2009; Blanco-Ulate et al., 2015), which in turn drive the biosynthesis of stilbenes, proanthocyanidins and anthocyanins, even in a white berry cultivar, such as Sémillon (Blanco-Ulate et al., 2015). In addition, Noble Rot (NR) (but not grey mold) induced different expressions of genes related to auxin and salicylic acid (up-regulated) and of one gene related to cytokinin (down-regulated). Two jasmonate *O*-methyltransferase isoforms were markedly induced both in the NR x Furmint interaction and in bunch rot inoculated Marselan berries (Pogány et al., 2022). In this study, NR development triggered the transcription of genes involved in the initial steps of the phenylpropanoid pathway, such as several isoforms of PAL and trans-cinnamate 4-monooxygenases (CYP). Their activation enabled the formation of key phenolic compounds, such as cinnamic acid and p-coumaric acid, which are the initial bricks of polyphenol biosynthesis. Remarkably, the activation of those genes was not limited to *Bc* in the NR form, as it is also shown to be a common transcriptional signature of grape berry cells elicited by *Bc* infection (Kelloniemi et al., 2015; Agudelo-Romero et al., 2013). Stilbene synthase (*STS*) isoforms were uniformly upregulated in both NR and bunch rot (BR) berries. Oppositely, the expression of two chalcone synthase-encoding genes (*CHS*) varied between NR and BR. Indeed, NR triggers a downregulation of *CHS*, which could justify the limited amounts of flavonols found in Sémillon berries affected by NR (Blanco-Ulate et al., 2015), whereas BR upregulated expression of these genes (Pogány et al., 2022).

**Figure 2:**
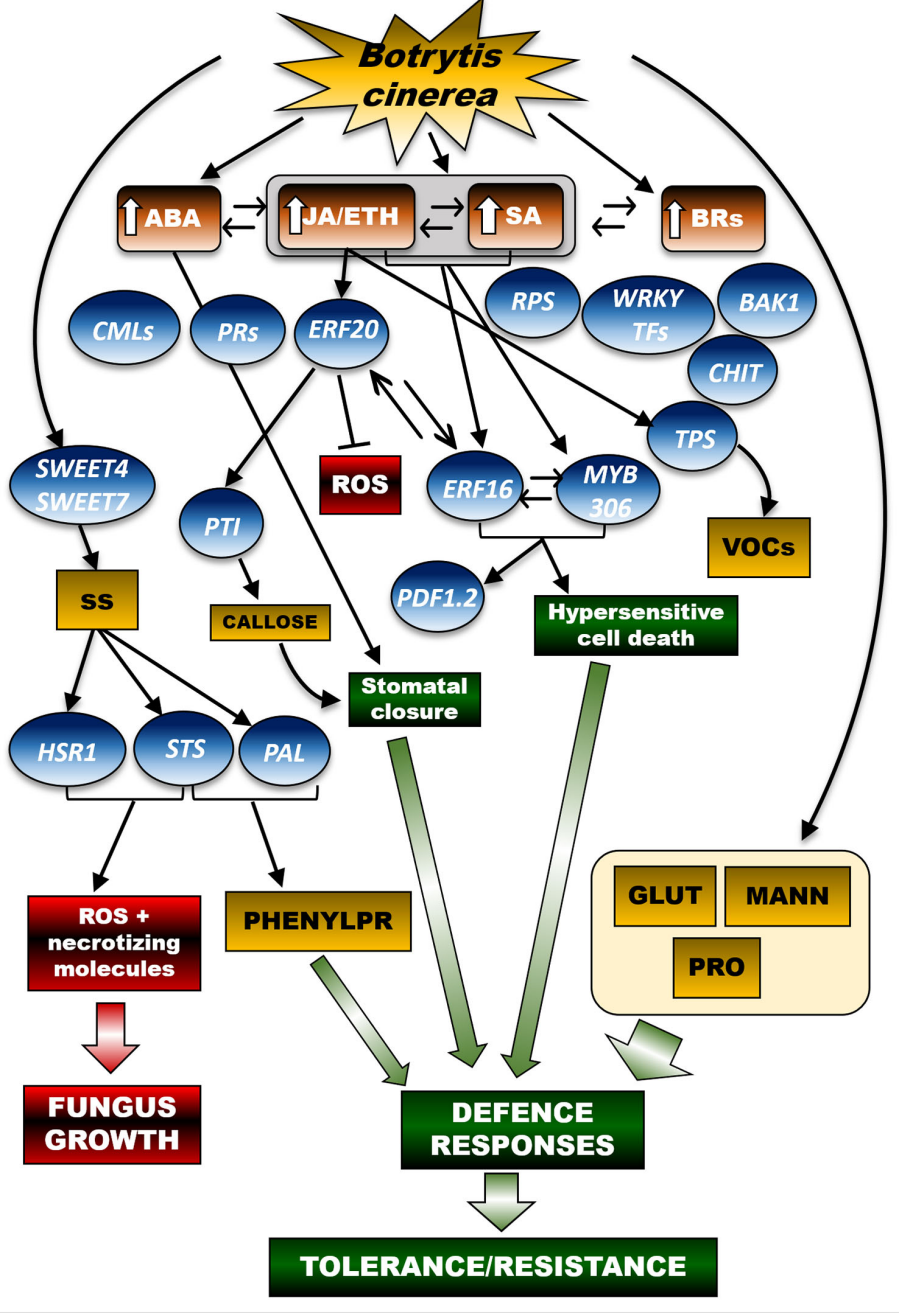
A schematic representation to display the networks of hormonal and molecular signals controlling defence secondary metabolic pathways in the grapevine leaf in response to *Botrytis cinerea (Bc)*. In young and adult grapevine leaves, tolerance/resistance responses to *Bc* involve the crosstalk of different hormonal pathways, namely ABA (abscisic acid), JA/ETH (jasmonic acidi/ethylene), SA (salicylic acid) BRs (brassinosteroids), in turn leading to activation of key molecular effectors of the plant defence system, such as PRs (pathogenesis-related proteins), RPS (disease resistance protein), CMLs (calcium-binding proteins), WRKY transcription factors, BAK1 factor (brassinosteroid insensitive 1 factor), ERF (ethylene responsive factor), and CHIT (chitinases). Infection-mediated metabolic biomarkers also include GLUT (glutathione) and osmoprotectants, such as MANN (mannitol) and PRO (proline). In parallel, defence mechanisms to *Bc* involve the activation of the sugar transporters SWEET4 and SWEET7 (Sugars Will Eventually be Exported Transporter 4 and 7), in turn enabling a leaf-specific increase in soluble sugar (SS) concentration. On one hand, SS accumulation turns on the transcription of *STS* (stilbene-synthase) and *HSR1* (hypersensitivity related protein 1 genes). The sugar signal triggers PAL-based biosynthetic pathways leading to the accumulation of key defence secondary metabolites (stilbenes). Upon *Bc* infection, JA/ETH and SA-responsive molecular cascades trigger the ERF20-mediated transcription of *PTI* genes, thereby facilitating plant immunity processes and inhibition of ROS (reactive oxygen species). Crosstalk between the JA/ETH and SA-based pathways enables ERF16 and MYB306, whose coordinated activity stimulates key immunity-related molecular effectors, such as the PDF1.2 factor (plant defensin factor 1.2), and hypersensitive cell death programs able to counteract the pathogen spread. Based on the infection timing, JA/ETH-responsive signals also promote the expression of TPS, thereby leading to the production of defence VOCs (volatile organic compounds). In the figure scheme, the arrows connecting the different signal effectors highlight activation of a specific molecular or metabolic pathway, whereas the blunt arrow points to inhibition of the downstream target(s). Crosstalk of hormonal or molecular signals is instead displayed by double arrows. The arrow beside the hormone acronyms refers to increase (↑) of that hormone accumulation. The thick green arrows point to the establishment of metabolic processes resulting from the activation of the upstream signalling pathway(s), namely defence responses of the plant (green boxes) or fungus growth-related processes (red boxes). Brown boxes refer to phytohormones, blue balloons indicate genes and yellow rectangulars refer to metabolites. SS, soluble sugars; VOCs, volatile organic compounds; ROS, reactive oxygen species; GLUT, glutathione; MANN, mannitol; PRO, proline; PHENYLPR, phenylpropanoids.

At the leaf level (**Figure 2**), the resistance mechanisms induced by *Bc* require the hormonal crosstalk of abscisic acid (ABA), salicylic acid (SA), ethylene (ETH), brassinosteroids (BRs) and jasmonic acid (JA) (Jia et al., 2022). Many genes are involved in the leaf’s resistance to *Bc*; they encode for pathogenesis-related proteins (*PRs*), disease resistance proteins (*RPS*), calcium-binding proteins (*CMLs*), *WRKY* transcription factors, brassinosteroid insensitive 1 (*BAK1*) factor, ethylene-responsive transcription factor (*ERF*), and chitinase 5. These genes were expressed differently in young and adult leaves. Furthermore, numerous infection-related metabolic biomarkers were identified, including glutathione and proline, which accumulated particularly in the young leaves, thereby suggesting a defensive activation based on leaf age (Pogány et al., 2022).

The activation of *ERF* transcripts following *Bc* infection was also demonstrated (Wang et al., 2018; Wang et al., 2020; Jia et al., 2022; Pogány et al., 2022; Zhu et al., 2022). In *Vitis amurensis*, *ERF20* (*VaERF20*) was induced by inoculation with *Bc*, both in leaves collected from the resistant Shuangyou cultivar (*V. amurensis*) and in those from the susceptible *V. vinifera* cv Red Globe. Accordingly, *Arabidopsis thaliana* mutants over-expressing (OE) *VaERF20* displayed enhanced resistance to *Bc* and a decrease in the concentration of pathogen-induced reactive oxygen species (ROS). These findings point to a specific response of tolerant genotypes to necrotrophic pathogens (Mengiste, 2012; Wang et al., 2018). Both salicylic acid (SA) and jasmonic acid/ethylene (JA/ET), responsive-defence genes were up-regulated in *VaERF20*-OE *Arabidopsis* plants inoculated with *Bc*. Pattern-Triggered Immunity (PTI) responses associated with increased expression of *PTI* genes, callose accumulation and stomatal closure were boosted in the transgenic lines with respect to wild-type controls. These data prove that *VaERF20* is involved in various signal transduction cascades and acts as an inducer of the plant immune response. In *Vitis amurensis*, *ERF16* (*VaERF16*), expressed during *Bc* infection and in response to ET and methyl jasmonate (MeJA) treatments, is also significantly up-regulated in berries (cv Shuang You; Zhu et al., 2022). Such results confirm a previous study reporting that *ERF16* was overexpressed in grapevine leaves inoculated with *Bc* (Zhu et al., 2019), thereby suggesting that *VaERF16* positively affects the plant’s immunity system against this pathogen. Although *Bc* is classified as a necrotrophic fungus, besides the JA/ET hormonal crosstalk, SA-mediated signalling (generally associated with biotrophic responses) also regulates the *Bc* × *Vitis* spp. interaction (Wang et al., 2020 *, Bc* × *Vitis quinquangularis*). Zhu and co-workers (2022) confirmed the involvement of the SA-based regulatory network in the *Vitis amurensis*-*Bc* pathosystem, indicating that in leaves, *ERF* genes contribute to the immune responses through both SA and JA/ET signalling pathways. Moreover, *ERF16* was suggested to regulate plant immune responses by interaction with other genes. In particular, *ERF16* interacts with *VaMYB306*, a homologue of *AtMYB30*, which is a positive regulator of the hypersensitive cell death programme in response to pathogen attack (Vailleau et al., 2002). Seventy-two hours after the *Bc* inoculum, the transcription of *VaMYB306* was induced up to 6‐fold in the leaves of *V. amurensis* ‘Shuang You’ in comparison with the non-inoculated controls. Accordingly, the leaves from *VaMYB306*-OE plants showed enhanced resistance against the pathogen whereas those from *VaMYB306-*silencing lines experienced an increased susceptibility. Moreover, both MeJA and ET enhanced *VaMYB306* transcription. This suggests that *VaMYB306* could promote resistance to *Bc* in a JA/ET signalling-dependent manner. Another important, recent finding elucidating the complex grapevine response to *Bc*, concerns the combined effect exerted by *VaERF16* and *VaMYB306*: together, they increased by 3.5-fold the promoter activity of *VaPDF1.2*, a key defence gene functioning downstream of the JA and ET signalling pathways (Zhu et al., 2022).

Based on the trigger effect exerted by JA on the accumulation of VOCs, including terpenoids, the influence of *Bc* infection on VOCs has been investigated by many authors. Recently, this subject has been thoroughly reviewed in grapevine (Lazazzara et al., 2021). In berries of Muller-Thurgau and Garganega artificially infected with *Bc*, a strong transcriptional activity of genes related to jasmonic acid and ethylene production was found (Lovato et al., 2019). These events were mainly directed to trigger the phenylpropanoid pathway, including the stilbenoid branch, as terpene synthase genes were highly expressed exclusively at the earliest stages of *Bc* infection. At later stages, a decrease in the expression of terpene synthase (*TPS*) genes was observed, indeed reducing the accumulation of terpenoids, in turn, and most likely enhancing the plant’s suceptibility to the patogen. A recent study on tomato plants has demonstrated that methyl-jasmonate-knockout-mutants plants showed a much lower TPS expression than wild-type controls, in turn resulting in a higher susceptibility to *Bc* (Cao et al., 2022). It was previously demonstrated that the timing of *Bc* infection could influence the regulation of *TPS* expression (Mendes et al., 2014). *TPS* transcripts were overexpressed early, following the fungal infection, and afterwards they were progressively downregulated. This information suggests that the *Vitis vinifera* × *Bc* interaction does not exclusively influence the higher or lower expression of *TPS*, but that these effects may co-exist, and a major role in defining their occurrence seems to be played by the moment at which the interaction is studied. A recent metabolomic analysis further revealed significant differences in the initial metabolic profiles of berries from two Greek *Vitis vinifera* varieties, Limnio and Roditis, which display high and limited tolerances to the pathogen, respectively. In response to the inoculation, the abundance of several osmoprotectants increased in the resistant Limnio cultivar, namely proline and mannitol, and some phenylpropanoids and metabolites associated with lignin biosynthesis (Tziros et al., 2022). Sugar transporter genes (*SWEET*s genes, Sugars Will Eventually be Exported Transporter) are also involved in the grapevine response to necrotrophic pathogens. In detail, *SWEET4* was upregulated in response to *Bc* infection in the leaves of *Vitis vinifera* line 40024, 72 and 96 hours after pathogen inoculation, together with two other isoforms (*VvSWEET2* and *VvSWEET7*), which were moderately induced (Chong et al., 2014). The *SWEET4* upregulation occurred concomitantly with a tissue-specific increase in sugar concentration. This probably facilitated the fungus’s nutrition and is associated with increased expression of *VvSTS* and *VvHSR*, higher accumulation of ROS, and higher accumulation of necrotizing molecules (Chong et al., 2014). Additionally, *SWEET7* expression was induced upon *Bc* (and *Erysiphe necator*) infection, probably to support the pathogen nutrition and to promote the grapevine response to the pathogen *via* production of defensive secondary metabolites (Breia et al., 2021). Accordingly, the local SWEET-mediated increase in sugar concentration could serve as a trigger for the observed activation of the phenylpropanoid pathway. Such strict relationship between sugar accumulation and phenylpropanoid pathway activation was long-time demonstrated in berries (Pirie and Mullins, 1976). It is therefore conceivable that the up-regulation of *SWEET* genes can not only support the pathogen growth, but it can also boost the grapevine’s defence metabolism through the activation of the PAL-mediated-downstream pathways. In the *Bc* (as NR) × grapevine interaction, carbohydrate-related enzymes were activated, particularly at the onset of NR development, during the transition from the phase I to II of berry growth. In line with what was observed for *TPS*, these findings reveal that the main structural changes in the plant’s defence metabolism occur early in the NR process (Hegyi et al., 2022).

### 3.2 Grapevine trunk diseases

The five main diseases recently referred to as GTDs are: *Botryosphaeria* dieback, *Eutypa* dieback, the esca disease complex, *Phomopsis* dieback and *Black foot* (Bertsch et al., 2013). However, other fungal genera and species, especially *Ascomycota* and *Basidiomycota*, have been found in association with GTDs, and it has also been demonstrated that the specific fungal association depends on climatic and geographical factors. *Ascomycota Eutypa lata* is the main causal agent of *Eutypa* dieback, and *Phaeomoniella chlamydospora, Phaeoacremonium minimum* and *Fomitiporia mediterranea* are the main causal agents of one or more of the syndromes associated with the esca complex. These fungal species are certainly the most widely diffused, though several other pathogenic fungi have been isolated from esca symptomatic plants (Mugnai et al., 1999; Surico, 2009; Del Frari et al., 2021). GTDs main symptoms include wood discoloration, necrosis and foliar symptoms. Strong manifestation of these symptoms induces profound alteration of the grapevine physiology, leading to shoot death, yield reduction, and the death of canes, spurs and even the vine. A progressive weakness of the viticultural agro-ecosystem can be ascribed to GTDs *per se* and to their interactions with vines in increasingly arid conditions. Another factor that significantly influences the GTD x grapevine interaction is the grapevine’s age. The GTD-associated fungal population inhabiting adult vines includes esca, *Botryosphaeria*, and *Eutypa*. Conversely, the main GTDs in young leaves are the Black foot disease, Petri disease or diebacks due to *Botryosphaeriaceae, Verticillium or Fusarium* as recently reviewed by Claverie and co-workers (2020). These authors proposed three conceptual models of the framework grapevine × GTDs × environment. The first describes the possible timing of events leading to leaf symptoms. Accordingly, foliar symptoms appear at the end of three steps (infection, colonization and symptom appearance) that can last from few months to several years, or can occur several times per year and on different parts of the plant, ultimately leading to yield reduction, berry quality loss, and wood destruction. The second model considers the grapevine × pathogen interaction and explains that the vine’s need to establish a defence response should be considered as a further sink for carbon demand. This sink becomes a competitor of shoot growth, floral induction, berry set, and berry ripening, thereby unbalancing the physiological status of the whole vine and seriously endangering the plant’s survival, sometimes leading to apoplexy. The third model provides a complex and holistic interpretation that adds to the grapevine × fungi relationship the microbiota and the environment. Since viticulturists design the vineyard establishment, including rootstock choice and agronomic and protection practices, they must be aware that their choices can also impact GTD spread, and in turn impact grapevine physiology.

The contribution of this review is mainly based upon the second and the third models. In a vineyard historically affected by Esca disease, beside the main Esca-associated fungi *P. chlamydospora* and *Fomitiporia* sp., five further genera (*Debaryomyces, Trematosphaeria, Biatriospora, Lopadostoma, and Malassezia*) were found in adult (19 years old) grapevines of Cabernet Sauvignon vines (Del Frari et al., 2019). Collectively, the authors identified 289 taxa, revealing the greatest fungal richness until now. However, 80% of these taxa displayed very low abundances (< than 0.1%) and high variability in their composition depending on factors such as plant age, agricultural practice, and environmental conditions. No positive, neutral or negative roles have been ascribed to them. Nevertheless, in the ongoing climate change scenario, one can speculate that these or other new taxa could emerge in the next years, and either become detrimental for viticulture or remain neutral (Corredor-Moreno and Saunders, 2020). Oppositely, they could acquire a positive role, if they are able to limit the development of more pathogenic *taxa*. The type of fungal species associated with the Esca disease in wood depends on the analysed organ (Del Frari et al., 2019) and on the *Vitis* species. Artificial inoculations of *Fomitiporia punctata* in Kober 5BB (*V. berlandieri x Vitis riparia*) led to low re-isolation percentages of this pathogen (8% of inoculated plants; Sparapano et al., 2000). Del Frari and co-workers (2019) did not find *Fomitiporia* sp. in the graft union point of 140 Ru (*V. berlandieri x V. rupestris*), albeit *Fomitiporia* sp. was well represented in all permanent wood of Cabernet Sauvignon. Such findings confirm the hypothesis that the *Vitis* genotype can profoundly influence the disease development. Interactions of the mycobiome with *Vitis* genotype, plant age, organs, and environment, clearly demonstrate that studies on singular interactions between grapevine with one or a few wood pathogens are limitative with respect to reality in a viticultural context. However, the biological understanding of the interplay that comes from these kinds of studies is fundamental to interpreting present and future grapevine/wood disease interactions on a broader scale, as well as at a productive level.

Modifications of plant metabolism occurring early during the disease development have been detailed, demonstrating that alterations become greater as soon as visible symptoms appear on the leaf (Valtaud et al., 2009). Mainly, an increase in leaf sugar concentration was observed together with *PAL* transcript activation and consequent polyphenolic accumulation in leaves. Changes in tannin content (increase) and glutathione concentration (decrease) in apparently healthy-looking leaves located on infected shoots were described at the beginning of the season. Furthermore, changes in glutathione *S*-transferase (*GST*) gene expression in the leaves of Ugni blanc collected from healthy and diseased grapevines in field conditions were assessed. At early stages of infection prior to the appearance of visible leaf symptoms, GST activity, amounts of *GSTU1*- and *GSTF2*-encoding genes and abundance of the GSTU1 and GSTF2 proteins were the highest. Afterwards, they progressively decreased during the season, as soon as visible leaf symptoms emerged (Valtaud et al., 2009). Moreover, in another study conducted on adult (>20 years old) Chardonnay grapevines grafted onto the 41B rootstock (*V. vinifera x V. berlandieri*) and affected by the leaf stripe form of Esca, an early induction of *GST1* expression was noticed in pre-symptomatic leaves, suggesting that early and transient activation of *GST1* could help the vine to detoxify fungal toxins (Magnin-Robert et al., 2011). In that study, an inverse regulation of photosynthesis- and defence-related genes was also observed. Similarly, a gradual, transient increase in *PAL*, *LOX* and *PR6* expression occurred in pre-symptomatic leaves in parallel to a decrease in the vine’s assimilation rates. Unexpectedly, the expression of those defence-related genes was inhibited in the green area of fully symptomatic leaves and no alteration was observed in the chlorotic area. These results suggest that grapevine may perceive some signals and react by triggering defence pathways before the appearance of foliar symptoms. This highlights the grapevine’s ability to activate its defence system promptly; however it is also evidence of reduced capability to promote strong defence reactions once the leaves become symptomatic. Previous studies demonstrated that the activation of *PAL* and *STS* genes involved in the biosynthesis of stilbenoids result in high amounts of resveratrol both in woody tissues (Amalfitano et al., 2000; Martin et al., 2009) and in leaves and berries of esca-affected vines (Calzarano et al., 2008). The GST pathway activation could be an early grapevine response to Esca disease, intended to distribute the pathogen-induced signals throughout the vine vascular system (Valtaud et al., 2009; Magnin-Robert et al., 2016). Amounts of fungal metabolites, plant metabolites (stilbenes) and hormones (ABA), and plant transcripts involved in pathogenesis and water stress response (e.g. *PRs*, *HSPs*, *SOD*, *GSTs*, *TIPs*, *NCED*), changed in apoplectic and pre-apoplectic grapevine tissues. Morevoer, carbohydrate, aminoacid and phenylpropanoid related-gene expression varied with grapevine age. For instance, young apoplectic grapevines had higher phenol concentrations in wood than esca-diseased plants, while asymptomatic wood of diseased plants showed higher stilbene concentration (Magnin-Robert et al., 2016). This knowledge, together with previous information on phenylpropanoid activation after different pathogen attacks may call for a shift toward either CHS or STS activation in grapevine tissue following biotic stress events. A recent paper (Khattab et al., 2021) proved that some lines of *V. vinifera sylvestris*, the ancestor of the cultivated *V. vinifera*, with different levels of susceptibility to *Neofusicoccum parvum* (associated with *Botryosphaeriaceae*-related dieback), accumulated higher amounts of stilbenoids, resveratrol and trimeric viniferins. The authors proposed that a stress signal, including a JA-mediated signal, triggered the activation of PAL, STS and JAZ in all genotypes, regardless of their level of susceptibility. Furthermore, while susceptible lines accumulated piceid (the glucosidic form of resveratrol, probably less efficient in limiting pathogens, due to its lower antioxidant activity), the resistant lines produced high amounts of non-glycosylated resveratrol and viniferins.

Time can influence the grapevine × GTD interaction: a time-dependent response of grapevine varieties to *P. chlamydospora* was found in a model study of cuttings of three grapevine cultivars, Merlot and Carignan - considered moderately susceptible - and Cabernet Sauvignon - considered highly susceptible - were let absorbing a culture filtrate of the fungus. Very early, the least susceptible varieties showed an overexpression of *PR*-proteins, *PAL* and *STS* genes, together with a higher concentration of stilbenoids, respect to highly susceptible Cabernet Sauvignon (Lambert et al., 2013). Overexpression of *PAL* and *STS* was also found in Cabernet Sauvignon cuttings inoculated with *P. chlamydospora* alone, with *Phaeoacremonium aleophilum* alone, or with the two pathogens together (Pierron et al., 2016). These authors noticed that other genes were also overexpressed, namely *PR10.3, TL, TLb, Vv17.3, STS8, CWinv, PIN, CAM, LOX*, and that all of them (with the exception of *LOX* and *CAM*) were up-regulated in response to the wounding caused during the pathogen inoculation. However, although this baseline noise was detected, it was clearly found that gene overexpression still differs based on perception of mycelium. Moreover, 48 hours after inoculation, the induction of *PAL* and *STS8* differed depending on the pathogen, revealing a higher over expression in the interaction of grapevine with *P. aleophilum* rather than with *P. chlamydospora* (Pierron et al., 2016).

Further important breakthroughs into the understanding of GTD × grapevine interaction were provided by Fischer and co-workers (2016). In the totally controlled conditions of the co-culture of *Vitis vinifera* L. *calli* (cv Gamay Fréaux) with *P. aleophilum* and *P. chlamydospora*, changes were observed in the fungus metabolism. Transcriptome reprogramming events were differently induced by the two fungi: *P. chlamydospora* triggered an increase in the expression of oxidoreductases, plant cell-wall degrading enzymes and detoxifying enzymes, whereas *P. aleophilum* activated only some genes encoding oxidoreductase, as well as heat shock and chaperon-like proteins, and enzymes involved in primary metabolism. Although the two fungi occupied the same ecological niche within the grapevine trunk, by predominantly developing within the xylem vessels and surrounding cell walls, they displayed different metabolic expressions (Fischer et al., 2016). However, regardless of the fungal species, the number of fungal genes differentially modulated during the grapevine’s callus formation was extremely low, probably because the plant cells did not cause extreme stress on the fungi and did not solicit very aggressive measures by the fungi. These findings are consistent with the observation that *P. aleophilum* and *P. chlamydospora* can be found in apparently healthy plants, that the outbreak of the disease may take several years to occur, and that young vineyards rarely show Esca symptoms.

In a study concerning the tripartite interactions among greenhouse-grown cv Cabernet Sauvignon at the state of 7-8 expanded leaves, *P. chlamydospora*, and the biocontrol-oomycete *Pythium oligandrum*, both the grapevine’s and the fungus’s metabolisms were affected. The biocontrol oomycete *Pythium oligandrum* shaped the expression of specific grapevine defence-related genes, and the *P. chlamydospora* + *P. oligandrum* association promoted JA/ET signalling pathways, particularly at early infection stages (Yacoub et al., 2020). Previous studies on *P. chlamydospora* demonstrated its pectinolytic capacity (Marchi et al., 2001), suggesting that the fungus can degrade pectin-rich pit membranes as well as deposits of gel/tylose that are secreted in vessels by the host in response to infection. Accordingly, histopathological studies confirm that *P. chlamydospora* mainly resides in the vasculature (Valtaud et al., 2009; Fleurat-Lessard et al., 2010) and can also spread within those vessels occluded by tyloses and gels, thanks to its pectinolytic activity. The grapevine’s need to synthesize occluding material to limit the fungus progression might reduce the energy resources otherwise used to establish active chemical defence responses, particularly in peri-vascular tissues. Therefore, for cultivars with xylem vessels of small diameter, like Merlot, vessel occlusion strategies could be quicker and easier, and consume less energy to restrict the spread of mycotoxins throughout the sap. On the contrary, in cultivars with wider vessels (e.g Cabernet Sauvignon) those reactions could be less efficient in hindering the fungus’s spread. In addition, in small vessel-cultivars, the ease of occluding the vasculature could favour the physical compartmentalization of fungi (Pouzoulet et al., 2014; Pouzoulet et al., 2020a). A recent study showed that in the nursery, three different grafting techniques adopted on three different *V. vinifera* varieties onto the same rootstock (Kober 5BB) allowed diverse levels of callus formation and vascularization of the grafting point (Battiston et al., 2022). Such results confirm the influence of the genotype on wood formation and response to pathogens. However, these authors did not find a strict relationship between vessel diameter/density and the potential susceptibility of the vine to GTDs. Ramsing and co-workers (2021) correlated 25 *Vitis* spp. rootstocks with DNA concentration of *P. chlamydospora* and *P. minimum*, demonstrating that some of them, such as *V. berlandieri* Resseguier 1 and Castel 6736 (*V. riparia* × *V. rupestris*), harboured significantly lower pathogen DNA concentrations in association with significantly narrower vessel diameters. Based on the rootstock genotype, the authors reported narrower vessel diameters for *V. berlandieri* × *V. vinifera*, *V. riparia*, *V.riparia* × *V*. *rupestris* and *V. rupestris.* The threshold for the identification of grapevine susceptibility to *P. chlamydospora* and *P. minimum* was associated with the pathogen DNA load. For instance, significantly higher *P. chlamydospora* DNA concentrations were detected for vessel diameters ranging from 55 to 64 μm and vessel area of 0.2–0.24 mm^2^. Conversely, a higher *P. minimum* titre was found for a xylem density of above 66 vessels/mm^2^ (Ramsing et al., 2021). Foglia and collaborators (2022) studied the vessel diameter of 24 red-berry varieties and 27 white-berry varieties, all grafted on Kober 5BB (*V. berlandieri* ×*V. riparia*), and showed that *V. vinifera* varieties display a wide variability in vessel diameters, ranging from 70-80 μm to around 160 μm. Interestingly, authors found an on-average higher vessel diameter in the white varieties respect to the red ones. However, they did not identify any clear correlation between vessel diameter and cultivar susceptibility to GTDs.

We were not able to find specific information about Kober 5BB vessel diameter, however we could for SO4, another hybrid of *V. berlandieri* × *V. riparia.* In 2016 Santarosa and co-workers classified this genotype as a vigorous rootstock (Santarosa et al., 2016). Ramsing et al. (2021) confirmed these features, also showing high vessel density and high level of vascularization.

Since European viticulture is mostly grafted, it is important to merge this fundamental information. Different *Vitis* species display different vessel diameters, which confer to the rootstock-scion combination different vessel embolization tendency: wider the vessel, higher the hydraulic conductivity, higher the vigour induced to the scion, but higher the tendency to embolize under drought. Moreover, grapevine vessel diameters depend on the water availability of the season, as morphological traits of the vascular system, such diameters, are known to present developmental plasticity that responds to environmental factors during the plant growth (Lovisolo and Schubert, 1998; Munitz et al., 2018; Pouzoulet et al., 2020b). Besides the hydraulic aspects tied to vessel dimensions and density, the higher/lower regulation of water transport under perivascular cell metabolic control in both root and shoot (Lovisolo et al., 2008a; Perrone et al., 2012; Chitarra et al., 2014) could be pivotal to understanding the GTD relationship with embolism repair strategies in grapevine. For instance, it can be hypothesized that *V. berlandieri* × *V. rupestris* rootstocks, which embolize less than *V. berlandieri* × *V. riparia* rootstocks, and also display a more efficient way to recover from drought embolisms (Lovisolo et al., 2008a), could be less susceptible to GTDs in field conditions. In turn, this feature could be linked to the mainly ABA-mediated metabolic control of water transport within the vasculature and vessel-associated cells (VACs). Cycles of drought events followed by fast recovery are progressively becoming more frequent in many viticultural areas worldwide (Patono et al., 2022) due to the pressure of climate change. Such conditions can increase xylem sap ABA concentration (Lovisolo et al., 2008b; Morabito et al., 2021) and ABA delivery from root to shoot. Since ABA signalling triggers the activation of *PAL* genes, we can suggest that the root-to-shoot ABA transport could regulate the accumulation of polyphenols in VACs, thereby acting as defence response to GTDs. This would therefore mirror what happens in leaves and fruits during fruit ripening, in line with what has been thoroughly investigated in grapevine × abiotic stress interactions (reviewed in Ferrandino and Lovisolo, 2014).

Such an intricate network of players adds to the changing vineyard environment and highlights the complexity of the study of the biological response of grapevine to GTDs; consequently, it also explains why successful protection practices addressed to control GTDs are still to be developed.

### 3.3 Phytoplasma infection

Phytoplasmas represent a group of phloem-limited wall-less microorganisms similar to bacteria, and taxonomically ascribed to the class of *Mollicutes*, which cause serious losses in productivity of many crops worldwide (Bendix and Lewis, 2018). In European viticulture, phytoplasma-associated diseases are commonly referred to as grapevine yellows; the most economically impacting are Flavescence dorée (FD) and Bois Noir (BN). Although FD outbreak occurred more recently than BN, vineyard damages due to its rapid spread were, and still are, heavier, especially in some countries like France and Italy. Symptoms associated with FD and BN infections are often indistinguishable visually; both typically display leaf yellowing or reddening depending on the grape cultivar, leaf downward curling, drying of inflorescences and bunches, fruit abortion, shortening of internodes, and lack of shoot lignification. Although no resistance to the two diseases has been detected yet, an increasing number of studies have reported different tolerance degrees according to specific cultivars (Eveillard et al., 2016) as well as cases of spontaneous symptom remission in field-grown vines (Morone et al., 2007; Santi et al., 2013; Landi et al., 2019). Additionally, it clearly appears that such physiological responses are often correlated with the accumulation of specific defensive secondary metabolites.

Mounting experimental evidence has demonstrated that among the first metabolic effects of FD and BN infection, there is a serious alteration in primary metabolic pathways and in the transport of hexose sugars, which relies, at the molecular level, on a wide transcriptional reprogramming of the functional gene categories linked to carbohydrate, photosynthesis, and energy metabolism (Hren et al., 2009; Prezelj et al., 2016; Pacifico et al., 2019; Pagliarani et al., 2020). These responses reflect the impaired physiological functions of infected vines, which display a reduction in the rates of assimilation, stomatal conductance and chlorophyll content (Vitali et al., 2013). Changes in photosynthate translocation inevitably cause a block of sugar export that, by strongly reducing phloem loading, causes the source-to-sink switch of leaves: a plant defence response also reported in presence of other leaf pathogens, such as fungi and viruses (Hayes et al., 2010; Chitarra et al., 2018). In parallel, this intense modification in sugar transport interferes and changes the secondary metabolite build-up; indeed, it is well established that mobilized sugars enter the shikimate pathway and the subsequent secondary metabolic reactions, particularly those tied to the phenylpropanoid biosynthesis (Ehness et al., 1997; Rolland et al., 2002). In fact, a common hallmark of phytoplasma infection is the conspicuous accumulation of flavonoid molecules in the leaves, particularly of anthocyanins, due to the sugar-mediated transcriptional activation of many flavonoid biosynthetic genes. Therefore, it has been proposed that the strong mobilization of sugars and antioxidant compounds can represent a biological strategy for counteracting pathogen spread and protecting plant tissues from further oxidative damage (Margaria et al., 2014).

Over the last years, knowledge of secondary metabolic modifications triggered by phytoplasma infection has been significantly advanced. This has highlighted interesting differences occurring in the profiling of specific classes of secondary metabolites that were put in correlation with genotype susceptibility, as well as with recovery mechanisms. In 2014, Margaria and co-workers, provided the first integrated metabolic and transcriptomic survey concerning the analysis of different compounds of the flavonoid biosynthetic pathway in FD-infected, recovered, and healthy leaves of the red-grapes Nebbiolo and Barbera, which are respectively reported to display low and high susceptibility to FD (Roggia et al., 2014). Although an increase in total anthocyanins did occur in both varieties following infection, anthocyanin concentrations were much higher in Barbera than in FD-infected-Nebbiolo samples, in accordance with the overall more intense reddening of the leaves of this cultivar. Nevertheless, infected Nebbiolo leaves showed an increase in quercetin glycosides with respect to kaempferol-derivatives, and a higher constitutive and FD-induced accumulation of low-molecular weight proanthocyanidins. These data, corroborated with different expression of key related genes and coupled with the observation that healthy and recovered Nebbiolo leaves were characterized by a higher basal content of proanthocyanidins than Barbera, led the researchers to hypothesize that those metabolic signatures could underpin the low susceptibility of Nebbiolo to the disease (Margaria et al., 2014). A thorough modulation of the phenylpropanoid biosynthetic pathway also typically occurs in vines affected by BN. Experiments recently performed by Negro et al. (2020) evidenced that BN-infected leaves of the red-grape Sangiovese accumulated high levels of total phenolics and flavonoids, concomitantly with a reduction in lignin concentration; they suggested this aspect was most likely a consequence of the diversion of the common precursors (hydroxycinnamic acids) towards the flavonoid pathway. The authors’ findings also revealed that increased levels of total anthocyanins in BN-positive samples were exclusively due to the predominant synthesis of cyanidin 3-O-glucoside, whereas BN-negative leaves accumulated the highest concentrations of quercetin.

It is, however, important to remember that some of the above-described responses are strongly dependent on the cultivar genotype; moreover, they could change based on the phytoplasma type and even on the specific phytoplasma strain (e.g. FDp C or D). For instance, in a study conducted on the highly FD susceptible cultivar Modra frankinja, Prezelj and co-workers (2016) found that compounds downstream of the proanthocyanidin branch, like epicatechin and catechin, accumulated greatly in infected samples. This is in line with what was found in infected Barbera vines (high susceptibility) studied in Margaria et al. (2014).

Intriguingly, besides depending on genotype and sanitary status, the overall quantity and partitioning of secondary metabolites could differ based on the portion of analysed leaf tissue, as demonstrated in infected, recovered and healthy plants of Nebbiolo and Barbera by comparing levels and profiles of target metabolites in whole leaves, leaf blades and leaf mid veins (Ferrandino et al., 2019). Although flavonols were constitutively more expressed in whole leaves of Barbera than of Nebbiolo, the separate analysis of leaf blades and veins revealed that FD-infected Nebbiolo samples had higher flavonol contents, particularly quercetin glycoside. The authors also found that hydroxycinnamate (HCTA) concentrations followed different genotype-dependent accumulation patterns, indeed their concentration was higher in entire leaves of Barbera than Nebbiolo, regardless of the sanitary status. Conversely, in Nebbiolo, increases in HCTA amounts were detected only in association with the phytoplasma presence and only when leaf veins and blades were analysed separately, also disclosing a higher percent-incidence of the caffeic acid ester. Similar accumulation patterns were also shown for flavan-3-ols, for which higher synthesis of the dimeric proanthocyanidin B1 was observed in Nebbiolo, together with a higher constitutive and FD-induced concentration of astilbin (dihydroquercetin-rhamnoside; Kedrina-Okutan et al., 2018) in veins. Such peculiar changes at the level of single phenylpropanoid molecules may contribute to enhancing the antioxidant capacity of Nebbiolo, thus making this cultivar more efficient at limiting the phytoplasma spread than Barbera (Ferrandino et al., 2019).

Similar considerations on the involvement of individual molecules in phytoplasma pathogenesis and genotype-mediated defence responses come from the analysis of stilbenoids. However, it has been emerging only recently that increases in some groups of these compounds could also elicit disease tolerance and/or recovery mechanisms to phytoplasma diseases. Pagliarani et al. (2020) reported the highest concentrations of total stilbenoids in FD leaf vein-enriched samples of cv Barbera, but they observed the presence of specific stilbenoids, such as viniferin, exclusively in the samples taken from recovered vines and consistently with the overexpression of many stilbene synthase-encoding genes.

Distinct biological functions of single stilbenoid categories in relation to a specific sanitary condition (i.e. infected or recovered) were also suggested in the presence of BN. For instance, Negro et al. (2020) found higher amounts of resveratrol in BN positive samples. Therefore, changes in the polyphenol profiles can mark the different susceptibility degree of grapevine genotypes to phytoplasma. Further information gained on additional genotypes with intermediate levels of susceptibility to FD or BN will be precious for elucidating to what extent the phenylpropanoid pathway can contribute to establishing specific defence responses in different *V. vinifera* cultivated varieties. Additionally, the survey carried out by Paolacci and co-workers (2017) proved that the production of distinct phenols, stilbenoids in particular, in association with either infection or recovery is achieved through deep perturbations in hormone signalling cascades, respectively involving salicylic (SA) and jasmonic (JA) acids. Such tight relationship between phytoplasma infection/recovery and hormone-based regulation of stilbene metabolism is an intriguing research matter (Dermastia, 2019) that has recently been further investigated by considering diversification of such signals depending on genotype-phytoplasma interaction and on the phenological phase (Dermastia et al., 2021). For instance, cv Zweigelt infected with ‘*Candidatus Phytoplasma solani*’ showed high induction of three transcripts of several genes that encoded for S-adenosyl-L-methionine:salicylic acid carboxyl methyltransferase, which catalyses the formation of the volatile ester methyl salicylate from salicylic acid, showing a peak in the early growing season, concomitant with the overexpression of genes encoding PR proteins. This allowed the identification of a salicylic-acid-dependent, systemic-acquired, resistance signalling. In addition, some genes of the auxin-responsive *GH3* gene family were up-regulated in the infected grapevines of cv Zweigelt. *Candidatus Phytoplasma solani* and FD induced the differential expression of several genes involved in jasmonate biosynthesis, generally showing opposite behaviour in the expression of lipoxygenases, so that the JA role in phytoplasma x grapevine interaction has still to be elucidated. Fifty-nine of the genes associated with ethylene metabolism were affected by *Candidatus Phytoplasma solani* infection of cv Zweigelt, with marked difference during the vegetative season: four genes were up-regulated more than 3-fold early in the season, whereas two genes of the ethylene signalling network were down-regulated later in the growing season. A similar trend was noticed for gibberellin 3-oxidase1 and for a proline-rich protein, which displayed more than 3-fold increase of expression early in the growing season, whereas an overall decrease in gibberellin oxidase gene expression was observed later. Finally, a strong down-regulation of two members of the brassinosteroid biosynthetic branch, both involved in sterol production, was observed in the late growing (Dermastia et al., 2021).

Unlike phenylpropanoids, phytoplasma-mediated changes charged to isoprenoid metabolic pathways have been much less investigated. Recently, novel insights into this subject have been reported by Teixeira et al. (2020) in vines of the Portuguese cultivar ‘Loureiro’ affected by FD. Following an integrated approach of targeted metabolomics and candidate gene expression analysis, the authors showed that FD infection significantly inhibited all the isoprenoid core metabolic pathways in infected leaves by strongly repressing the key genes associated with chlorophyll, carotenoid, quinone and tocopherol metabolism. Notably, it also emerged that despite the accumulation of many carotenoids, leaf ABA levels increased following FD infection. This is an interesting finding, which deserves further investigation, as it was not accompanied in that survey by upregulation of related biosynthetic genes. Additionally because other authors have reported ABA increases but in association with the recovery, instead of infected sanitary condition (Pagliarani et al., 2020).

## 4 Concluding remarks and perspectives

All investigations above-described well underline the great complexity of the molecular dynamics governing the build-up of grapevine secondary metabolites in response to altered climate scenario, biotic stress, and genotypes.

Despite the available information, further research efforts should be made to gain a complete picture of the interconnection of primary and secondary metabolic pathways that regulate genotype-specific quality traits at the berry level, particularly those depending on hormonal and sRNA networks. For instance, many open questions remain in the elucidation of epigenetic marks that prime grapevine adaptability to environmental stresses. A deeper knowledge of all those subjects would be undoubtedly helpful for improving viticultural practices and also developing sustainable breeding approaches. In the ongoing scenario of climate change, the main goal for grape growers is to adapt available vineyard management strategies to increasingly challenging conditions, based on the support provided by the research and technical communities. Clone and rootstock choices represent indeed a precious resource for helping in the mitigation of environmental stress effects, both abiotic and biotic. One must be able to mitigate the effects of climate alterations by boosting the grapevine’s defence system while preserving fruit quality traits, and to do this, novel, sustainable viticultural practices are needed in the short term (as recently reviewed by Gutiérrez-Gamboa et al., 2021).

Additionally, although modern genome editing techniques could offer promising cutting-edge tools for improving cultivar resilience and productivity (Bailey-Serres et al., 2019), the use of such technologies in viticulture is still prohibited in the EU. In addition, the commercialisation of resistant grapevine hybrids is limited and still under discussion, with issues raised related to the preservation of geographical identity of grape cultivars and consumer’s appreciation (Pedneault and Provost, 2016). The commercialisation of new varieties released by breeders is slow because of the long juvenile period of grapevines and of the need of field experiments (Brault et al., 2022). Therefore, the possibility to adopt and adapt viticultural practices able to mitigate the numerous issues arisen by climate change represents the most valuable strategy on the short-term period.

However, a further emerging strategy to face the negative effects of climate alterations and spread of diseases and pests relies on the exploitation of existing grapevine biodiversity. An effective and timely response to the current global and rapidly changing challenges will indeed be feasible only if the variability of grapevine genetic resources is as large as possible. Rediscovering and re-evaluating minor but important local varieties can offer interesting applications in terms of sustainable breeding approaches in viticulture. Those are indeed varieties naturally adapted to the conditions of the areas where they had been domesticated, and that can produce wines with unique qualitative and sensory characteristics, able to mirror the character, typicity and success of the wines that historically attract the consumers. A deeper knowledge of the physiological processes underlying the tolerance of these minor, autochthonous grapevine genotypes to abiotic and biotic stresses will be valuable for increasing the resilience of the whole viticultural system (Wolkovich et al., 2018). Such an approach is therefore particularly promising as it could offer a pratical solution for adapting the ‘tomorrow’ viticulture to future environmental conditions (Antolín et al., 2022).

## Author contributions

AF and CP contributed to the conception of the review. AF, CP and EP-A wrote the first draft of the manuscript. AF and CP wroteand over-viewed the final version. All authors contributed to thearticle and approved the submitted version.
